# 

**Published:** 1922-07

**Authors:** 


### HOSPITAL and HEALTH

REVIEW

Established 1886 — No. 1852. Vol. LXXI.

New Series. Vol. 1. No. 10 — July 1922 — Price Sixpence

CONTENTS.

| | PAGE |
|---|---|
| NOTES AND COMMENTS :— | |
| *Health.*—The Increase of Cancer—The Water Shortage—New Health Legislation—The Tuberculin Test in Cattle—Imperial Research—The National League for Health, Maternity and Child Welfare—The Education of the Deaf Child—Teachers and Sick Children—Free Meals for School Children—The County Nursing Muddle—Home Nursing for Insured Persons—The Danger of Copper Poisoning—The Public Health Services | 273 |
| *Hospitals.*—A Self-supporting Hospital—A Hospital Bequest Disputed—Institutional Care of the Aged—Making History at a Mental Hospital—The Spirit of the Staff—Hospital Portrait Galleries | 278 |
| THE PUBLIC HEALTH : INTERVIEWS WITH LOCAL AUTHORITIES. I.—LIVERPOOL | 280 |
| THE HEALTH MINISTRY'S PROGRESS. By the Rt. Hon. C. Addison, M.D., M.P. | 283 |
| LUNACY REFORM. By H. J. Norman, M.B., Ch.B. | 285 |
| THE ROYAL INSTITUTE OF PUBLIC HEALTH : PLYMOUTH CONGRESS | 286 |
| THE MONTH IN PARLIAMENT | 287 |
| THE BRITISH HOSPITALS ASSOCIATION CONFERENCE | 288 |
| THE VOLUNTARY HOSPITAL : A RETROSPECT AND A PROSPECT. By W. Thelwall Thomas, M.B.E., Ch.M., F.R.C.S. | 290 |
| OBITUARY : DR. W. H. R. RIVERS | 293 |
| EUGENICS AND PUBLIC HEALTH | 294 |
| THE NEED OF MATERNITY HOMES | 296 |
| PREVENTIVE AND RESCUE WORK | 297 |
| HOSPITAL FINANCE | 298 |
| HOSPITAL AND OTHER NEWS | 300 |
| CORRESPONDENCE :— The British Medical Association and the Voluntary Hospitals—A Hospital's Water Supply—Approved Societies and Hospital Treatment — Help for Armenia | 301 |
| APPOINTMENTS | 303 |
| OFFICIAL PUBLICATIONS : DIARY OF THE MONTH | 304 |

